# New and Emerging Viruses of Blueberry and Cranberry

**DOI:** 10.3390/v4112831

**Published:** 2012-11-06

**Authors:** Robert R. Martin, James J. Polashock, Ioannis E. Tzanetakis

**Affiliations:** 1 USDA-ARS Horticultural Crops Research Laboratory, Corvallis, OR 97330, USA; 2 USDA-ARS, GIFVL, 125A Lake Oswego Rd. Chatsworth, NJ 08019, USA; Email: james.polashock@ars.usda.gov; 3 Department of Plant Pathology, Division of Agriculture, University of Arkansas, Fayetteville, AR 72701, USA; Email: itzaneta@uark.edu

**Keywords:** *Vaccinium*, virus detection, disease

## Abstract

Blueberry and cranberry are fruit crops native to North America and they are well known for containing bioactive compounds that can benefit human health. Cultivation is expanding within North America and other parts of the world raising concern regarding distribution of existing viruses as well as the appearance of new viruses. Many of the known viruses of these crops are latent or asymptomatic in at least some cultivars. Diagnosis and detection procedures are often non-existent or unreliable. Whereas new viruses can move into cultivated fields from the wild, there is also the threat that devastating viruses can move into native stands of *Vaccinium* spp. or other native plants from cultivated fields. The aim of this paper is to highlight the importance of blueberry and cranberry viruses, focusing not only on those that are new but also those that are emerging as serious threats for production in North America and around the world.

## 1. Introduction

The genus *Vaccinium* belongs to the *Ericaceae* (heath family). The family has more than 3,500 species growing in all latitudes, from the tropics to the polar regions. A common feature among members of the family is that they grow in acidic, many times, nutrient-deprived soils [1]. Of the more than 400 species in the genus *Vaccinium*, highbush, lowbush and rabbiteye blueberries (*V. corymposum* L.; *V. augustifolium* Ait. and *V. ashei* Reade, respectively) and cranberry (*V. macrocarpon* Ait.) are of high economic importance. Largely due to recent press regarding the health benefits of fresh fruit consumption [2,3,4], including blueberries and cranberries, the industry has been rapidly expanding and blueberries in particular are now grown across the globe [5]. This change has come with the cost of introducing and spreading virus diseases and driving disease epidemics. The cost of virus diseases is massive in perennial fruit crops such as blueberry and cranberry that require a large initial investment to prepare, plant, and establish fields to the point of maturity and full productivity. Even when mature, intensive maintenance is needed to sustain productivity, but once established, fields can remain productive for many years. Some cultivated cranberry beds in New Jersey and Massachusetts are reported to be over 100 years old [6]. Blueberry fields are generally not as long-lived, but can easily be maintained for 30 years or more. 

Like most plants, *Vaccinium* spp. are known to harbor viruses belonging to several different families. Virus diseases are accompanied by a variety of symptoms ranging from being visually asymptomatic to plant death. In addition, symptoms can vary between cultivars, regions, production systems and years. Viruses can also be latent for years or not express at all in a nursery setting, further allowing widespread distribution. Virus diseases, particularly of blueberry, have become a major problem in recent years not only because of the expansion of the industry and the change in climate patterns, but also because of lack of grower awareness of the problems that can emerge when propagating non-certified material. Because of the rapid increase in blueberry plantings in recent years, in many cases growers choose to propagate from field plants that could be harboring asymptomatic infections at the time cuttings were taken. This has led to the emergence of several diseases in new areas or to major increases in virus incidence where there was minimal virus presence [7].

This review not only aims to provide up-to-date knowledge of the viruses that infect blueberry and cranberry but also give insight into the measures that need to be taken to control the spread of virus diseases and avoid virus epidemics, so as to be able to grow these crops in a sustainable and profitable environment. As is the case with virus diseases of any vegetatively propagated crop, the most critical control measure is the production and planting of stocks free of targeted pathogens. In the U.S., the programs for producing certified planting stocks of vegetative fruit crops has received a tremendous boost with the development of the National Clean Plant Network (NCPN). NCPN has as its’ mission the production and maintenance of the initial starting materials for certification programs that are free of targeted pathogens. Thus, the program aims to introduce cultivars of plants that are needed or desired for fruit production, including new materials developed outside of the U.S. to protect current production and the environment from the introduction of potentially damaging pathogens. The structure of this review is primarily based on the mode of transmission of the viruses as this is the most important factor for the development of an efficient and effective management strategy for the viruses and the diseases they cause.

## 2. Nepoviruses—Nematode and/or Pollen-Borne

### 2.1. Blueberry Latent Spherical Virus

Blueberry latent spherical virus (BLSV) was isolated recently from highbush blueberry in northern Japan [8]. Although the virus is readily transmissible to herbaceous hosts, and causes symptoms on indicator species that include *Chenopodium quinoa* and *Nicotiana benthamiana* it appears to be asymptomatic in the nine highbush cultivars evaluated [8]. BLSV is most closely related to *Peach rosette mosaic virus*, a subgroup C *Nepovirus *that also infects blueberry*. *The genome organization of BLSV is typical of members of subgroup C, with the replication-associated polyprotein in RNA 1, and the movement and coat proteins of the virus encoded in the single polyprotein of RNA2. The only difference in BLSV, as compared to other subgroup C viruses is that it has a serine instead of a cysteine protease encoded in RNA 1 [8]. Given the recent discovery of the virus, its geographic distribution and modes of transmission are still unknown, whereas detection protocols are under development.

#### 2.1.1. Blueberry Leaf Mottle Virus

**Figure 1:**
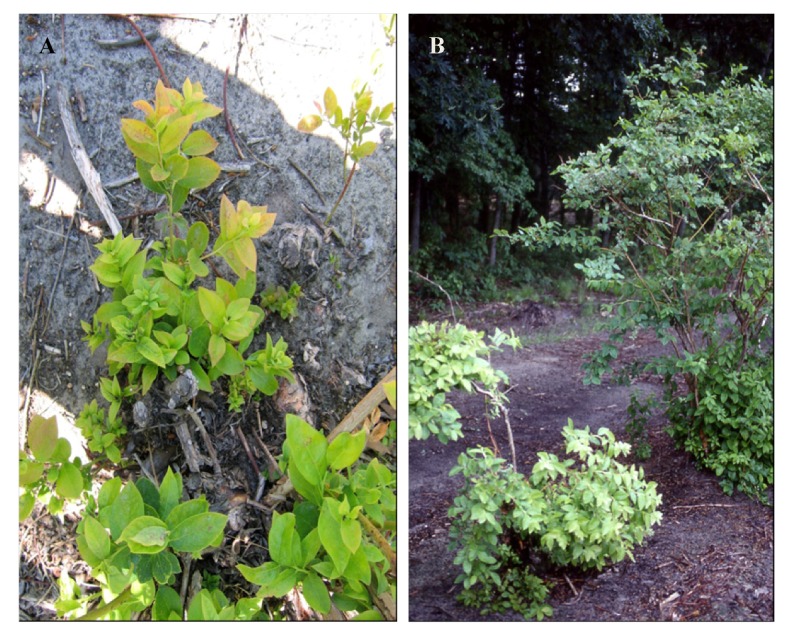
Symptoms of *Blueberry leaf mottle virus*: (**A**) small, pale green, rosetted and narrow leaves; (**B**) pale green, stunted Jersey bush (on left) (Photos courtesy Annemiek Schilder, Michigan State University).

Blueberry leaf mottle disease was first described in 1977 in Michigan and its presence is limited to this state and parts of eastern Canada [9] although extended surveys have been conducted in Arkansas, Oregon, Washington and British Columbia, Canada [10]. Affected leaves are smaller, distorted and show mottling symptoms (Figure 1). Affected bushes develop stem dieback, are stunted, and yield only a fraction of their healthy counterparts [11]. Symptom intensity is cultivar-dependent with northern highbush blueberry developing most severe symptoms [12]. Ramsdell and Stace-Smith [13] purified a virus from infected material. The virus proved to be the causal agent of the disease and was given the name *Blueberry leaf mottle virus *(BLMoV). The physicochemical properties of the virus and the weak cross-reactivity of *Grapevine Bulgarian latent virus* (GBLV) to BLMoV antisera pointed to the relationship of the virus to members of the genus *Nepovirus* [13]*.* Partial sequence of the two RNAs of the virus placed BLMoV in subgroup C of the genus [14,15]. Although the virus belongs to the genus *Nepovirus*, nematode or aphid transmissions studies have proven unsuccessful. The virus is readily transmissible by pollen, primarily via honeybee movement and seed [16] similar to another virus in the C subgroup, *Cherry leaf roll virus* [15]. For many years there has been confusion on the identity of BLMoV and GBLV. This has been cleared up after the molecular characterization of GBLV [17]. The two viruses are closely related but are clearly different species. BLMoV can be detected serologically and by RT-PCR; however, there is no data on its population structure, a concern for reliable testing. These data indicate that BLMoV is thus far only found in North America, knowledge that has obvious implications in quarantine and certification schemes.

#### 2.1.2. Peach Rosette Mosaic Virus

Rosette mosaic disease of peach was first described in the early 1900s [18]. It took several decades before a virus was associated with the disease [19]. The virus, *Peach rosette mosaic virus* (PRMV), early on was only found in stone fruit trees, grapevine and a few weed species such as dandelion. It was first reported in highbush blueberry in 1981 when northern highbush blueberry was planted into fields previously occupied by PRMV-diseased grapevines [20]. Symptoms include uneven leaf distribution, leaves that are smaller than usual with obvious deformations. No data are available on the effect of the virus on other types of blueberries or yield. Similar to BLSV and BLMoV, the virus belongs to subgroup C of the genus *Nepovirus *[15,21]. PRMV is unique among all nematode transmitted viruses as it can be transmitted by different nematode genera, namely *Xiphinema *and *Longidorus* [22,23]. The distribution of the virus is limited to the area around the Great Lakes of North America (Michigan, Ontario and New York) where the virus has only been found in a few fields. As in the case of BLMoV, PRMV can be detected serologically and by RT-PCR; however there is only information available for a single virus isolate. Given the unknown of virus diversity, extra precautions need to be taken when interpreting detection results. Reports of the virus in Europe and the Middle East need to be further evaluated as the virus was only detected using a single technique and the readings appeared very weak [24,25]. For certification and quarantine purposes, the virus should only be considered present in the areas aforementioned.

### 2.2. Necrotic Ringspot Disease [Tobacco Ringspot Virus (TRSV) and Tomato Ringspot Virus (ToRSV)]

**Figure 2:**
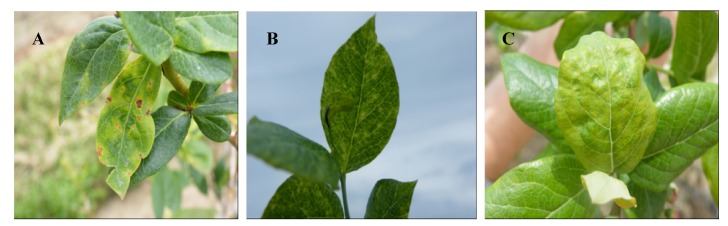
Symptoms of necrotic ringspot disease in blueberry: (**A**) *Tomato ringspot virus* in blueberry showing necrotic spots and leaf distortion; (**B**) Chlorotic mosaic symptoms on plant infected with ToRSV; and (**C**) Mosaic on young leaves of plant infected with *Tobacco ringspot virus*.

Necrotic ringspot disease was first reported in blueberry in 1960 in New Jersey [26] and was associated with TRSV in 1963 [27] and later reported in Arkansas, Connecticut, Illinois, Michigan, New York, Oregon, New Brunswick and Chile [28,29,30]. ToRSV was first reported in blueberry in Washington in 1972 [28] and subsequently identified in blueberries, in Indiana, Michigan, New York, Oregon, Pennsylvania, Canada, and Chile [9,28,29,30]. Necrotic ringspot disease (Figure 2) has been used synonymously for TRSV infection in blueberry, as this was the virus first associated with the disease. However, symptoms of ToRSV (Figure 2) are very similar to those caused by TRSV and the only way to differentiate the viruses in blueberry is to carry out diagnostic assays. TRSV and ToRSV are vectored by the same nematode, *Xiphinema americanum*, and disease symptoms in the field often appear as oval foci typical for nematode transmitted diseases. Both viruses cause a slow, steady decline in bush productivity in susceptible cultivars, including ‘Collins’, ‘Elliott’, ‘Jersey’, ‘Pemberton’, ‘Rubel’, and in several halfhigh blueberry clones in New Brunswick [9]. In some cultivars, infection with TRSV or ToRSV can lead to plant death. ‘Bluecrop’ appears to become infected very slowly with ToRSV, but it is highly susceptible to TRSV [28]. Necrotic ringspot symptoms of TRSV or ToRSV have not been reported in lowbush or rabbiteye blueberry. 

TRSV and ToRSV are typical of members in subgroups A and C, respectively, in the genus *Nepovirus* [14]. They contain two positive-sense genomic RNA molecules that are encapsidated in spherical virions of ~28 nm. Both have broad host ranges, are pollen- and seed-transmitted in some hosts with varying degrees of efficiency up to 100%, features that allow them to persist in the environment. TRSV and ToRSV can be detected serologically with ELISA and by RT-PCR; however, there are significant strain differences in both viruses, and one must take care to ensure that an appropriate test is used. TRSV is very common in *Rubus* spp. [31] in the southeastern U.S. and one would expect it to become an emerging problem in blueberry in that region as production expands there. 

TRSV and ToRSV are often difficult to detect in blueberry due to uneven distribution in the plants [29], seasonal differences in virus titer in leaf tissues and varying titers of virus in different parts of the plant. These viruses spread very slowly from an infection source and can be controlled by removing affected bushes, plus an additional 3–4 bushes beyond the symptomatic plants, taking care to remove as much of the root system as possible. The area should then be treated with a nematicide prior to replanting. If planting into a site that has widespread distribution of TRSV or ToRSV and the vector nematode present, then control will require removal of the entire field, followed by fumigation and replanting, fallowing the field and keeping it weed free, or planting a crop that is a non-host for these viruses [32]. This latter situation can happen if a site is not tested for the presence of *X. americanum* and the viruses prior to planting, since the viruses and nematode have broad host ranges and are symptomless in many hosts. 

## 3. Ilarviruses—Pollen-Borne

### 3.1. Blueberry Shock Virus (BlShV)

**Figure 3:**
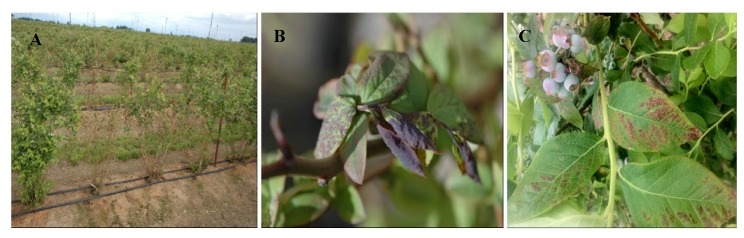
*Blueberry shock virus* symptoms: (**A**) Leaf and flower necrosis in cv. ‘Liberty’ showing many symptomatic and asymptomatic bushes in a production field; (**B**) Close up of leaf necrosis in cv. ‘Bluegold’; and (**C**) Leaf reddening in recovered plants that has been observed in several cultivars in years with prolonged cool conditions in Oregon.

Blueberry shock disease was first observed in Washington in 1987 and initially confused with blueberry scorch caused by *Blueberry scorch virus *[33]. However, plants affected with shock produced a second flush of leaves after flowering and the plants appeared normal by late summer except for the lack of fruit. Also, after 1–3 years the affected plants flowered and fruited normally and did not exhibit any additional symptoms [34]. However, during the long cool spring conditions of 2010, 2011 and 2012 plants of several cultivars that had recovered from the shock symptoms exhibited a leaf reddening in the Pacific Northwest (PNW; Oregon, Washington and British Columbia) (Figure 3C). The impact on yield, if any, with the leaf reddening symptoms is still unknown. The virus is pollen-borne and infection only occurs during the bloom period, as determined with trap plants placed bi-weekly in diseased fields throughout two growing seasons [35]. The year after infection, bushes exhibit a “shock reaction” where the flowers and foliage blight in the early spring just as the plant is in full bloom (Figure 3A,B). The blighted tissues fall from the bush and a new flush of leaves develops during the summer. By harvest time, infected bushes look nearly normal except for the absence of fruit. Fruit loss is correlated with the extent of blighting in the spring. In some bushes, only one or a few branches will show symptoms whereas in others the entire bush blights. Bushes, where only partial blighting occurs, will usually show symptoms the following year on previously symptomless wood. BlShV has been identified throughout the PNW, and more recently in California, Nova Scotia, Canada, Pennsylvania, New York [36] and Michigan [37]. 

BlShV is a member of subgroup 3 of the genus *Ilarvirus*. It is detected readily by ELISA or RT-PCR from flower buds early in the season and leaf tissue through August in the PNW. Control should be focused on not introducing the virus to new production areas on nursery stock. Once the virus is present in a field, removal of infected plants based on symptoms or diagnostics will slow the spread of the virus but not completely prevent further spread. There are several reasons for this: (1) The virus is unevenly distributed in blueberries the first year or two after infection and field testing can easily result in false negatives; and (2) The virus is pollen-borne and the flowers open enough to start releasing pollen before symptoms appear, thus transmission occurs before it is obvious a plant is infected. The recommendation in the PNW is to let the virus run its course through a field realizing that there will be a 1–2 year crop loss in the process. This is less of a loss than removal of a field and replanting, where it may be 4–6 years before a planting is back in full production. It is unknown whether plants will recover in a similar manner in other production areas where the growing conditions are less favorable. Thus, an approach similar to that used in Michigan, where the entire field was removed as soon as the virus was detected, is recommended if the virus is detected in a new production area.

### 3.2. Tobacco Streak Virus

*Tobacco streak virus* (TSV) was first reported in cranberry in 2001 [38]. The virus was discovered during a routine screen of plant material imported into Scotland. The material originally tested was shipped from New Jersey and the source plots in New Jersey remain infected. It was later noted that vine material in question was brought to New Jersey from Wisconsin. Subsequent limited testing of vines in Wisconsin indicated varying degrees of infection [39] and the prevalence of virus in Wisconsin is still unknown. The infected material showed no symptoms and it is unclear whether the virus has any deleterious effects under certain conditions or in certain cultivars. TSV belongs to subgroup 1 and is the type member of the *Ilarvirus *genus in the family *Bromoviridae*. Although there are both immunological and molecular tests available for the detection of the virus, the extreme diversity observed among isolates needs to be taken into consideration when choosing a detection protocol. TSV has been reported to be thrips transmitted through incidental mechanical inoculation as the insects feed on the plants and infected pollen is introduced into the wounds [40]. This mode of transmission has not been demonstrated in cranberry. Seed transmission may occur at very low incidence, but thus far, seedlings from crosses using infected pollen have tested negative [39]. 

## 4. Aphid-Borne

### 4.1. Blueberry Scorch Virus

A previously undescribed blight of highbush blueberry was observed since the early 1970s in the Sheep Pen Hill area of Burlington County, New Jersey [41]. The symptoms were described as a blighting of both flowers and new vegetative growth just prior to full bloom. The cause at the time was unknown, but it was noted to be actively spreading to all cultivars grown in the area. The disease, known locally as ‘Sheep Pen Hill Disease’, was noted to be of economic importance as it could lead to total fruit loss and it was cautioned that the disease could potentially spread through plants propagated for sale. A disease with similar symptoms was described in 1980 as affecting the cultivar Berkeley near Puyallup, Washington [42]. Both diseases were shown to be graft transmissible and virus particles were found in the leaves of affected plants [42,43]. The disease etiology was characterized in both states and was shown to be caused by distinct strains of new carlavirus [44,45,46]. The disease became known as Blueberry scorch and the causal agent was thus named *Blueberry scorch virus* (BlScV). The virus particles are flexuous rods approximately 14 × 650 nm encapsidating the monopartite positive-sense single-stranded RNA genome of about 8.5 Kb [47,48]. 

**Figure 4:**
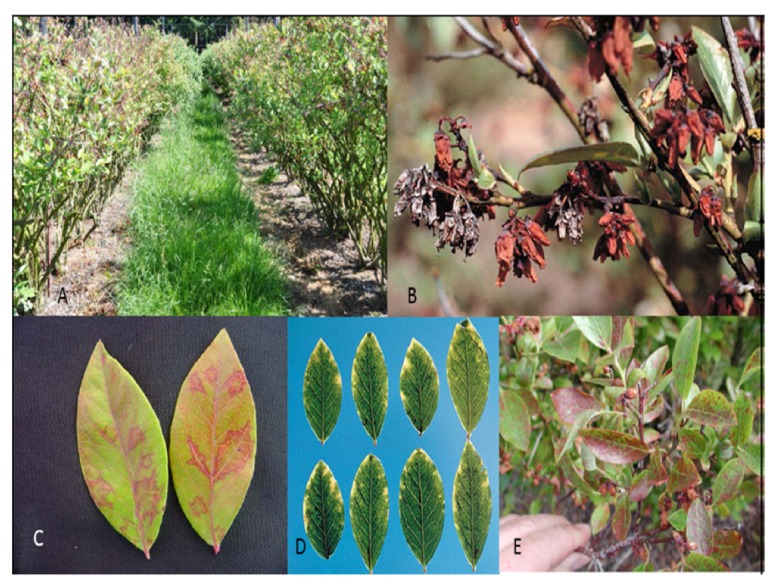
*Blueberry scorch virus *symptoms: (**A**) Blueberry cv. ‘Elliott’, necrotic flowers and leaves in foreground, healthy bushes in background; (**B**) Blueberry cv. ‘Berkeley’ showing flower necrosis and retention, silvery flowers are from previous year, brown flowers from current year; (**C**) Line and oakleaf patterns; (**D**) Chlorotic leaf margins in cv. ‘Stanley’; E. Leaf reddening in cv. ‘Concord’.

Symptoms vary depending on virus strain and cultivar from asymptomatic to severe blighting of flowers, young leaves and twig dieback. In addition to these symptoms, leaves of infected bushes sometimes show marginal chlorosis or a red line pattern in late summer and fall (Figure 4). The blighted flowers often fall off soon after blighting, but can remain on the bush throughout the season and through the next dormant season (Figure 4B). Plants with severe blighting bear little or no fruit and take on a scorched appearance (Figure 4A). Virus infections can be latent for many years, depending on cultivar, and severity of expression can vary from year to year. Affected bushes of some cultivars can be productive for many years, whereas others decline quickly over a few years and eventually die. This variation in symptom expression has made field diagnosis difficult and growers are reluctant to remove infected bushes that still bear fruit. The disease has been reported in New Jersey, Massachusetts, Connecticut, Michigan, Oregon, Washington, and British Columbia, Canada [49]. The disease has also been reported in Germany [50], Italy and found in a single plant in the Netherlands [51]. The virus is spread primarily by aphids in a non-persistent manner [52]. BlScV has also been reported from American cranberry (*V. macrocarpon*) and black huckleberry (*V. membranaceum*), but those infections were asymptomatic [53,54]. An aphid control program can help limit field spread. Aphids can also transmit the virus to *Chenopodium quinoa* and *C. amaranticolor *[48] as well as *N. occidentalis* [55]. Mechanical transmission has been successful using infectious transcripts [56]. Blueberries are typically asexually propagated by rooted cuttings. The method of pruning plants to the ground and allowing regrowth for cutting wood production does not allow symptom expression in infected plants. Thus, distribution through infected nursery stock is also an important mode of dissemination [57]. It is imperative that all plants be purchased from certified nurseries that test material with state-of-the-art protocol, able to detect all known virus strains so as to minimize the danger of infected material and the movement of virus and disease into areas where it is absent. 

### 4.2. Blueberry Shoestring Virus

**Figure 5:**
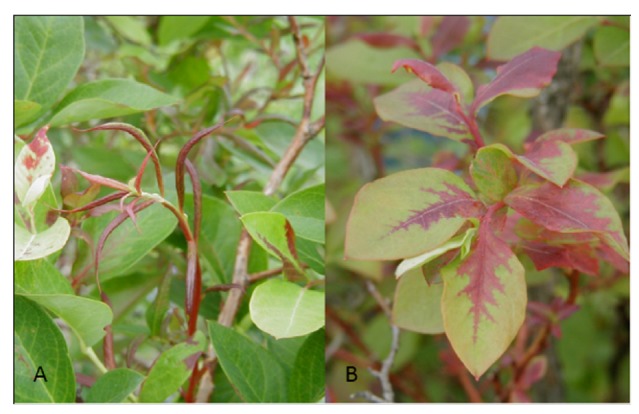
*Blueberry shoestring virus* symptoms: (**A**) Narrow strapped leaves that may be reddish in color, note not all leaves exhibit symptoms; and (**B**) Oakleaf pattern of reddening on normal shaped leaves (Photos courtesy Mark Longstroth Michigan State University).

The disease was first described in 1950 [58]. Expanding twigs develop red streaking in the spring, discoloration that normally disappears as the season progresses. Symptoms on leaves vary significantly from narrow, pointy leaves to distorted, fully developed blades that may develop an oakleaf pattern (Figure 5). There is normally red discoloration among the veins, symptoms that may affect one or more areas of the leaf, to the point that it covers the majority of the blade. Symptoms vary in intensity depending on the environment and there have been reports were bushes can be asymptomatic for several seasons [59]. Symptoms often are only observed on parts of a plant rather than on all leaves. In severe cases, the disease leads to extensive losses, because of yield reduction and production of unmarketable fruit [60].

Varney [59] provided the first evidence that shoestring disease is caused by a graft-transmissible agent. A virus, *Blueberry shoestring virus* (BSSV), was proven to be the causal agent of the disease after Lesney *et al.* [61] purified the virus and inoculated healthy plants that developed typical diseased symptoms a few months post inoculation. Although BSSV was confirmed as the causal agent was described 35 years ago, it is still understudied. Ramsdell [58] studied the physicochemical properties of the virus, including size, composition and molecular mass of the coat protein. Those properties indicate that BSSV is member of the genus *Sobemovirus* and it is recognized as a member of that genus by the International Committee on Taxonomy of Viruses [62]. BSSV is the only approved member of the *Sobemovirus* genus that is transmitted by an aphid in an efficient manner that can reach 28% in inoculation studies with 30 aphids (*Illinoia pepperi*) that had an inoculation access period of one hour [63]. All types of blueberry are susceptible to the virus but infection rates appear to be higher in certain northern highbush cultivars [64]. As in several of the viruses described here, detection tests available today are based on a single virus isolate and additional research is needed to improve the detection spectrum to as many isolates as possible. 

## 5. Vector Unknown

### 5.1. Blueberry Latent Virus

A new disorder was observed in 2002 in Oregon and Washington in the U.S. and British Columbia, Canada. Highbush blueberry set but dropped virtually all fruit when about 5 mm in diameter. The possibility that a virus was associated with the disorder was examined and dsRNA was isolated from more than 20 affected and several asymptomatic plants. A single molecule of about 3.5 kb was present in the majority of affected plants and controls [65]. The molecule was not associated with the disorder but its presence in the vast majority of the plants tested led to its further characterization. The dsRNA belongs to a virus, provisionally named Blueberry latent virus (BBLV), and will probably be the type member of a new taxon of dsRNA viruses [66,67]. The genome organization of the virus, lacking a movement protein, suggested that it can only move by cell division. Another interesting feature of BBLV is its presence in high percentages in all blueberry germplasm tested from both the east and west coast of the United States as well as Japan [66,68]. Almost 50 isolates from the U.S. and Japan have been sequenced, partially or completely, and the population structure appears extremely stable as diversity does not exceed 0.5% among all isolates. Aerial and soil transmission studies have been performed without success. These data, in combination with the genome structure and extreme genome stability of BBLV, advances the idea that its movement is limited to seed and/or pollen and experiments with three northern highbush and one rabbiteye blueberry cultivar showed 100% seed transmission. It is expected, and given the high incidence of BBLV in all tested germplasm, that the virus is present wherever North American germplasm is grown. The prevalence of the virus should not be of major concern though as no symptoms have been observed on several highbush cultivars that have been monitored for almost 10 years.

### 5.2. Blueberry Mosaic Virus

**Figure 6:**
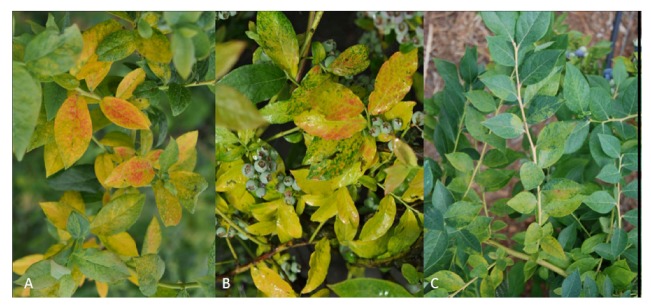
Blueberry mosaic symptoms in cultivars chlorosis, reddening and mosaic symptoms observed in many cultivars: (**A**) ‘Bluecrop’; (**B**) ‘Blueray’; and (**C**) ‘Legacy’.

Blueberry mosaic disease was first reported in the 1950s when experiments proved that it is not a genetic disorder but is rather caused by a graft-transmissible agent [69]. Mosaic is the most noticeable of all blueberry diseases. Symptoms include mottling and mosaic patterns of bright yellow to pink to red (Figure 6). Usually symptoms are apparent on only a few leaves on a bush, although, and depending on the season, they may be absent or cover the majority of the bush. The effect of the disease has not been studied in detail but some reports indicate that infected material has noticeable reduction in yield, and in addition the fruit is of poor quality and may ripen late [69]. Many northern highbush (*V. corymbosum*) cultivars develop symptoms [70]. No symptoms have been observed in other *Vaccinium* species other than *V. vacillans *(a lowbush dryland blueberry; [69]. It is important to note that there was no detection test available until recently [71] that would help determine whether the agent does not infect other blueberry species or causes asymptomatic infections.

There have been several attempts to identify the causal agent including reports of a viroid-like agent found in diseased plants [72]. The viroid was never proven to be the causal agent of the disease and only recently was the putative causal agent fully characterized [73]. All symptomatic plants collected from Arkansas, Michigan, New Jersey, Kentucky and Oregon were infected with a new member of the negative-sense, single-stranded RNA genus *Ophiovirus. *

The disease seems to be static in most areas but in recent years there have been observations of movement to adjacent bushes, suggesting that the vector is soil-borne as proven in the case of other ophioviruses [74]. The disease has been observed in several areas in North America whereas there are reports of limited distribution in New Zealand, Europe, South Africa, Argentina and Chile [75]. With the development of detection methods, it will be feasible to access the presence of the virus in blueberry production areas around the world and determine its effect on production.

### 5.3. Blueberry Necrotic Ring Blotch Virus

**Figure 7:**
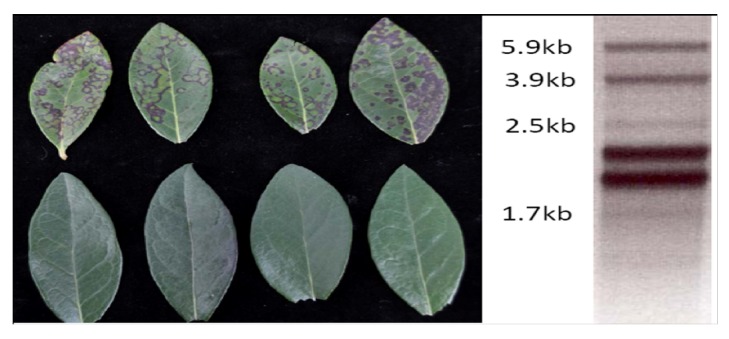
Blueberry necrotic ring blotch: (**A**) Virus symptoms in blueberry cultivar ‘Star’, upper row, symptomatic leaves showing range of necrotic rings and spots, lower row, healthy leaves; and (**B**) dsRNA extracted from symptomatic plants, the two bands between 1.7 kb and 2.5 kb, represent Totiviruses.

A new disease named blueberry necrotic ring blotch, was first observed on southern highbush blueberry in 2006 in Georgia in the U.S., and has since been observed in the southeastern quadrant of the U.S. including the states of Florida, Mississippi, North Carolina and South Carolina. The disease has not been observed in the northern highbush blueberries (*V. corymbosum*) or native rabbiteye blueberries (*V. virgatum*) in the region. The symptoms initially appear as distinct necrotic rings with green centers (Figure 7A), but as the rings coalesce they can be confused with symptoms caused by several blueberry fungal pathogens. In severe cases the disease progresses to premature defoliation of bushes and initially was thought to be caused by septoria leaf spot, which can also cause premature defoliation. The necrotic rings are observed on the upper and lower surface of the leaves, in contrast to BBRV, which causes rings that usually are only observed on upper leaf surfaces and occasionally on young green shoots. 

Investigations into the possibility of a viral etiology began with extraction of dsRNA from symptomatic and healthy leaf tissues. Six dsRNA bands were observed consistently from more than 10 sources of diseased tissue (Figure 7B), four of these were absent in unaffected tissue. The four dsRNAs were sequenced and found to have several unique features [76]. The most surprising was the presence of two helicases, which is unique among all known viruses. Additionally, these two helicases represent very different lineages suggesting that Blueberry necrotic ring blotch virus (BNRBV) is the result of a recombination event during a mixed infection in some host, not necessarily blueberry [76]. Additional support for creation of this virus by a recombination event is unique genome organization compared to its most closely related relative, *Citrus leprosis virus* (CiLV). CiLV has two RNAs compared with the four present in BNRBV. It appears that RNA 1 of CiLV is divided into RNA 1 and RNA 2 of BNRBV whereas RNA 2 of CiLV is divided into RNA 3 and RNA 4 of BNRBV. Thus, BNRBV likely represents a new virus genus [76]. *Hibiscus green spot virus* also shows similarities to BNRBV but has three genomic segments and triple gene block type of movement proteins in contrast to the 30K-superfamily type of CiLV and BNRBV.

The virus is detected readily by RT-PCR as there was a perfect correlation between symptomatic and asymptomatic tissue and RT-PCR positive and negative results, respectively, in over 60 isolates tested from multiple states. Future plans include testing that will address symptomless infections in rabbiteye, northern highbush, and additional cultivars of southern highbush blueberries.

Based on aa homologies with CiLV it is likely that BNRBV is transmitted by an eriophyid mite, and experiments to test this hypothesis are underway in Florida and Georgia [77,78]. In greenhouse trials, plants that tested-free of BNRBV developed symptoms in less than 3 weeks when placed adjacent to infected plants, whereas plants of the same cohort maintained in a greenhouse without any infected plants did not develop symptoms [78]. 

There is strong evidence for host resistance or tolerance, as some southern highbush blueberry cultivars are often found to exhibit extensive symptoms (*i.e.*, ‘Star’, ‘O’Neal’, and ‘FL 86-19’), whereas others on the same farm or field can be symptomless. Although spread through vegetative propagation appears to be limited, suspect plants should not be utilized for propagation through hard- or softwood cuttings. Plants produced in tissue culture from source plants that have tested free of BNRBV may help to reduce the initial introduction of this disease since they are protected from transmission by an aerial vector during much of the plant increase propagation cycles. 

#### Blueberry Red Ringspot Virus

**Figure 8:**
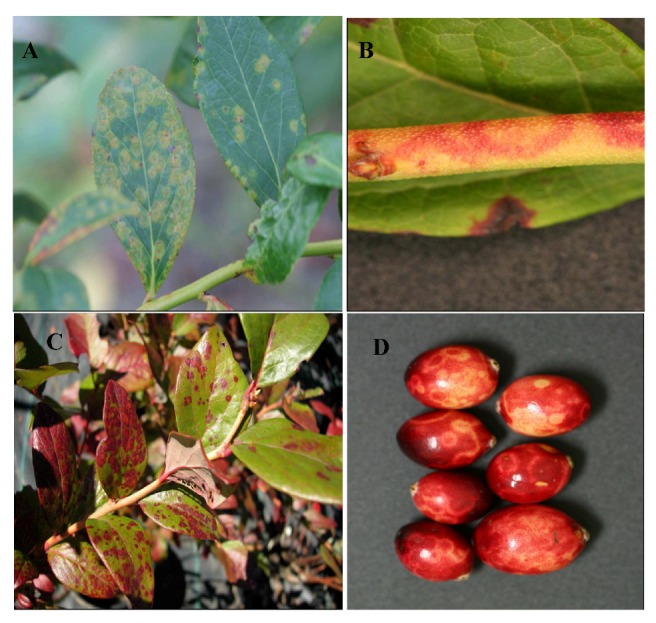
Symptoms of *Blueberry red ringspot virus*: (**A**) Early season leaf spots; (**B**) Red ringspots on young twigs; (**C**) Late season ring spots on leaves; and (**D**) Ringspots on cranberry fruit.

Red ringspot was first described as a disease of highbush blueberry in New Jersey in 1950 [79]. The disease was shown to be graft transmissible and presumed to be caused by a virus. The symptoms appear as red rings on green stems (Figure 8B) and as red rings with pale green centers 2–3 mm in diameter or round red spots that can coalesce into blotches on older leaves in late summer (Figure 8C). The rings on leaves were traditionally thought to be visible only on the upper surface of the leaves and this was used as a diagnostic character, but some cultivars exhibit the rings on both sides of the leaves. Occasionally, reddish rings appear on the developing green fruit, but are usually not apparent when fruit is fully ripe. Infected bushes of the cultivar ‘Ozarkblue’ exhibit deformed fruit that are not marketable [80]. Many infected cultivars appear to bear a full crop. However, a limited study in Michigan reported a 25% crop loss in infected plants of the cultivar ‘Blueray’, but impact on yield in other cultivars is to be documented [81]. A similar disease was reported to occur in American cranberry [82], but in cranberry the leaf symptoms are limited to small red splotches and the rings on the fruit are light colored on the reddening berries late in the season (Figure 8D). The causal agent in blueberry was confirmed to be a virus in the family *Caulimoviridae* in the *Soymovirus* genus and designated *Blueberry red ringspot virus* (BRRV). Virus particles are 42–46 nm in diameter and the circular double-stranded DNA genome is 8.3 Kb [83]. The virus is easily detected by PCR from symptomatic tissue. Detection in asymptomatic leaves is unreliable, but scrapings of green bark from current-year stems can provide reliable detection. Antibodies for this virus are not yet available. Cranberry is infected by distinct strains of the virus [84]. Vectors for the BRRV, as with all members of the genus *Soymovirus*, are yet to be identified, although the virus is actively moving in infected fields in the eastern U.S. The disease has been reported in blueberry in New Jersey, Michigan, North Carolina, Georgia, New York, and Connecticut, [28]. The disease has also been reported in Japan [85], Korea [86], Poland [87], Slovenia [88], and Czech Republic [89]. Since symptoms on soft wood cuttings are variable among cultivars and symptoms on hardwood cuttings may not be visible, infected plants may inadvertently be used as source material for propagation. Thus, spread through propagation of infected plants is also a source of distribution. Significant work on the virus population structure in the U.S. and areas around the world has led to the development of sensitive and reliable detection protocols that can be used for virus screening and minimize the mayhem that can be caused by the propagation and distribution of symptomless material [84]. 

### 5.4. Blueberry Virus A

**Figure 9:**
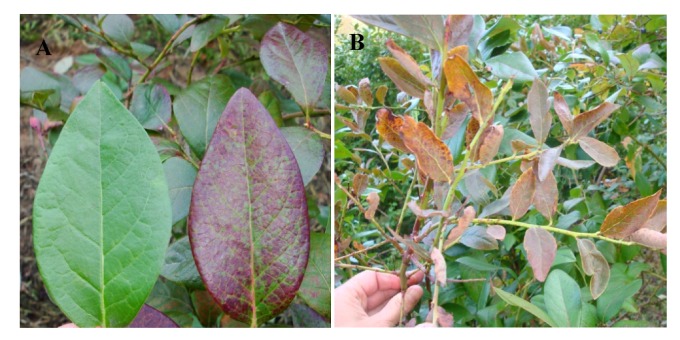
Symptoms of blueberry bronze leaf curl disease: A. Leaf exhibiting mid season reddening, healthy leaf on left, symptomatic leaf on the right; B. General bronzing with some upward curling of leaves (Photos Courtesy Annemiek Schilder, Michigan State University).

A new disorder named blueberry bronze leaf curl was observed in 2009 in Michigan in the U.S. Symptoms were observed in cultivars ‘Jersey’, ‘Bluecrop’, ‘Elliott’, ‘Duke’ and ‘Pemberton’ [90]. Plants exhibiting the disorder developed a bronze to red leaf color in mid-summer with some leaves curling upward (Figure 9). DsRNA extracted from symptomatic leaves was cloned and partially sequenced suggesting a novel virus in the genus *Closterovirus* was present in affected plants. Detection primers were developed, yielding amplicons of the new virus only from symptomatic plants [91]. A closterovirus, designated Blueberry virus A, from highbush blueberries in Japan recently has been sequenced and the entire genomic sequence is available in GenBank (accession # NC_018519.1, Isogai and Yoshikawa). Sequence comparisons between the U.S. and Japanese isolates showed 99% identity at the amino acid level, suggesting the same virus is present in Michigan and Japan. In Japan, the virus has been detected in the cultivars ‘Spartan’, ‘Sierra’, ‘Bluecrop’ and ‘Coville’ and there were no symptoms associated with the virus infection [92]. This suggests the possibility that the symptoms observed in Michigan may be due to mixed infections.

## 6. Conclusions

It has been more than 15 years since the last review of blueberry viruses [27]. In this period there have been new viruses and diseases described whereas additional information on the epidemiology of known viruses has accumulated, knowledge that we aimed to capture in this review. As with the case of other berry crops, blueberry production has expanded to new areas, not only in North America, the cradle of vaccinium production but also around the world. Now, blueberry is grown on all continents except Antartica, from Europe, Asia, Australasia, Africa and South America. The major shift and expansion of blueberry production has led to new disease challenges. Blueberry is a prime candidate for problems caused by new and reemerging viruses for several reasons: (1) Many of the viruses that infect blueberry have a limited distribution compared to the areas where blueberry production occurs; (2) Production of this crop is expanding rapidly worldwide; (3) Plants are in high demand and in many cases cuttings for propagation are taken from fruiting fields without knowledge of their virus status; (4) As plantings are established in new areas the crop is being exposed to new viruses and vectors; (5) The reaction of new cultivars, which are deployed rapidly and widely, lack information on their susceptibility and reaction to viruses and other pathogens; and (6) Many countries do not have certification programs in place for this crop. All of the above reasons suggest that efforts to minimize the movement of viruses in planting stocks through effective certification programs will go a long way to safeguarding the blueberry industry. Plants are often sold across production regions, countries and continents, providing many opportunities for virus spread over long distances in planting stock. Development of certification programs to enhance the quality of the planting stock in terms of virus infection should be a high priority and where possible harmonization of the certification programs will facilitate safe and efficient movement of blueberry plants. There are a limited number of viruses that impact blueberries, but as the crop is grown in more regions it is expected that additional viruses will be detected in blueberries. A recent example of this is the occurrence of BNRBV in the southeastern U.S., which occurred only a few years after the industry there began to expand rapidly. Also, once introduced into the southeastern U.S., *Blueberry red ringspot virus* appears to spread much more rapidly there than in other regions where it has been reported, suggesting the presence of a more efficient vector. 

Changes in cultural practices, changes in climate and reduced or altered pesticide use may all lead to increased virus incidence through indirect impacts on vector populations. As new cultivars are released, they may be more sensitive to virus infections and exhibit more severe symptoms and reduced yields. Alternatively, they may be tolerant of virus infection and facilitate movement of viruses in plants simply because they are symptomless carriers of a virus. Changes in climate likely will result in altered ranges of many virus vectors, such as, whiteflies, aphids, thrips, mealybugs, eriophyid mites *etc.* leading to changes in virus distribution. With the large scale of plant (ornamental and crop plants) movement internationally, introduction of new vectors into a production region can dramatically change the epidemiology of a virus disease (*i.e*., vine mealybug introduction to California greatly increased the impact of Grapevine leafroll viruses; expansion of the glassy winged sharpshooter into California increased the impact of Xylella on grape production). Once a virus is introduced into a new region eradication efforts can be very expensive (*i.e*., *Plum pox virus* eradication efforts after its introduction in Pennsylvania has cost the state more than 53 million dollars [93].

Blueberries are an expensive crop to establish, requiring extensive site preparation, irrigation, pruning and 3–5 years to bring into production. The cost of certified plants is relatively small compared to all of the other costs associated with getting a planting into production, yet using virus infected plants could lead to a completely unproductive planting, or at best a planting that takes longer to get into production and will have reduce yields over the life of the planting. Given the importance of the quality of mother plants and certification schemes that will minimize the movement of infected material, our group is working to provide material of the highest quality possible to the end user. Through the National Clean Plant Network we are working to eliminate all known viruses from propagation material, while developing sensitive and reliable tests for all blueberry viruses based on several isolates collected from multiple states in the U.S. and countries around the world. In addition, we study emerging diseases attributed to graft transmissible agents. This work aims to identify the agents and develop detection protocols that would allow for early detection and minimize movement within the indigenous area but also reduce the possibility of movement into areas where diseases are absent. As a systems-based approach to blueberry virus diseases we are also developing harmonized certification schemes among all blueberry production states in the U.S. that will allow for the movement of the highest quality propagation material to all areas where blueberry is grown. 
